# New challenges for microRNAs in acute pancreatitis: progress and treatment

**DOI:** 10.1186/s12967-022-03338-2

**Published:** 2022-05-04

**Authors:** Wence Zhou, Shi Dong, Zhou Chen, Xin Li, Wenkai Jiang

**Affiliations:** 1 The First School of Clinical Medicine, Lanzhou University, Lanzhou, 730000 Gansu China; 2 Department of General Surgery, The First Hospital of Lanzhou University, Lanzhou, 730000 Gansu China

**Keywords:** microRNAs, Acute pancreatitis, Pathogenic mechanism, Molecular markers, Treatment

## Abstract

Acute pancreatitis (AP) is a common clinical abdominal emergency, with a high and increasing incidence each year. Severe AP can easily cause systemic inflammatory response syndrome, multiple organ dysfunction and other complications, leading to higher hospitalization rates and mortality. Currently, there is no specific treatment for AP. Thus, we still need to understand the exact AP pathogenesis to effectively cure AP. With the rise of transcriptomics, RNA molecules, such as microRNAs (miRNAs) transcribed from nonprotein-coding regions of biological genomes, have been found to be of great significance in the regulation of gene expression and to be involved in the occurrence and development of many diseases. Increasing evidence has shown that miRNAs, as regulatory RNAs, can regulate pancreatic acinar necrosis and apoptosis and local and systemic inflammation and play an important role in the development and thus potentially the diagnosis and treatment of AP. Therefore, here, the current research on the relationship between miRNAs and AP is reviewed.

## Introduction

Acute pancreatitis (AP) is a very common acute disease of the digestive system [1] that is characterized by necrosis of pancreatic acinar cells and local and systemic inflammatory reactions [2, 3]. The incidence of AP is 13–45 per 100,000 people and is increasing [4]. The total mortality rate of AP is approximately 5%. When AP develops into severe acute pancreatitis (SAP), its mortality rate is as high as 20–40%, which seriously endangers people's lives and health [5–7]. Early AP can be cured by combination therapies, such as analgesia, nutritional support and protease inhibitors. However, without timely intervention, rapid development of AP will lead to SAP resulting in serious complications and even systemic multiple organ failure, which endangers the patient’s life. Because of the limited efficacy of conventional therapies and the lack of effective targets for treatment of AP, the prognosis of patients is often poor [8]. With the rapid development of high-throughput sequencing technology, researchers have found that microRNA (miRNA) intervention can change related physiological functions, causing inflammation cell infiltration, autoimmune diseases, cancer and other diseases [9, 10]. The role of miRNAs in inflammation provides a new direction for the treatment of AP. Targeting miRNAs to influence the progression and treatment of AP is a current research hotspot. To this end, this review discusses the research on miRNA mechanisms of action in AP and the potential use of miRNA for AP treatment, providing a theoretical reference for AP diagnosis, prognosis evaluation and targeted therapy.

## miRNAs and inflammation

As small noncoding RNAs, miRNAs are approximately 19–25 nucleotides in length and plays an important regulatory role in epigenetics. Mature sequences are mostly located in the introns, exons or pre-mRNA introns of noncoding RNA [11]. By targeting the 3'UTR of target genes, miRNAs control the mRNA translation process or accelerate the degradation of mRNA, ultimately regulating the expression of target genes [12]. Studies have shown that a single miRNA regulates multiple signalling pathways in the human body by targeting different mRNAs, including phosphatase and tensin homologue (PTEN), nuclear factor kappa-B (NF-κB), wingless/β-catenin (Wnt/β-catenin) and Janus kinase/signal transducer and activator of trans (JAK/STAT). miRNAs are widely involved in various cell activities in organisms, including cell development, differentiation, metabolism and apoptosis [11], and plays an important role in the occurrence and development of many diseases, such as inflammation, kidney injury and tumours [13].

Studies have found that inflammation is involved in the occurrence and development of many diseases in the human body, such as liver cirrhosis, pancreatic cancer, diabetes and rheumatoid arthritis [14–16]. Inflammatory cells and inflammatory factors are involved in the occurrence and development of inflammation, but the relevant molecular mechanisms regulating inflammation are still unclear. With the understanding of miRNA function, miRNAs have been found to play an important role in the production of inflammatory factors and inflammatory cells. For example, Let-7adf promotes the inflammatory response, metabolic activity, and interleukin (IL)-6 production by M1-type macrophages by regulating Tet methylcytosine dioxygenase 2/the deubiquitinating enzyme A20 (TET2/A20) [17, 18]. miRNA-93 can mediate the Toll-like receptor 4 (TLR4)/NF-κB signalling pathway to reduce the production of the inflammatory factors tumour necrosis factor-α (TNF-α), IL-1β and IL-6; reduce inflammation; and improve cell apoptosis [19]. In addition, some miRNAs play proinflammatory roles, such as miR-34a [21], miR-27a [22], miR-200a [23], miR-495-3p [24] and miR-124-3p [25], while others exert anti-inflammatory effects, such as miR-21 [20], miR-138 [26], miR-342-3p [27], miR-873a-5p [28], miR-146a [29], miR-542-3p [30], miR-193b-3p [31], miR-140-5p [32] and miR-27a-3p [33]. A summary of studies of miRNAs that affect the production of inflammatory cells and inflammatory factors is shown in Table 1. The involvement of miRNAs in inflammation-mediated processes may allow intervention in the progression of inflammatory diseases and effectively improve the prognosis of patients. The unique mechanism of miRNAs in inflammation is expected to provide novel therapeutic targets for rapidly developing inflammatory diseases, such as AP.

**Table 1:** Summary of studies of miRNAs that affect the production of inflammatory cells and inflammatory factors

miRNAs	Expression	Targets	Effect on inflammation	Inflammation-related cells or factors	References
miR-let-7a/let-7d/let-7f	Up	Tet2, Lin28a/Sdha axis	Promotion	Macrophages, IL-6	[17]
microRNA let-7	Up	A20	Promotion	Macrophages, TNF, IL-1β	[18]
miR-93	Up	TLR4/NF-κB	Suppression	TNF-α, IL-6, IL-1β	[19]
miR-21	Down	PDCD4/NF-κB	Suppression	Macrophages, TNF-α、IL-6	[20]
miR-34a	Up	KLF4	Promotion	Macrophages, TNF-α, IL-6, IL-1β, and MCP-1	[21]
miR-27a	Down	TLR4/MyD88/NF-κB	Promotion	TNF-α, IL-6, IL-1β	[22]
miR-200a	Down	Keap1/Nrf2	Promotion	TNF-α, L-1β	[23]
miR-495-3p	Up	IL5RA	Promotion	TNF-α	[24]
miR-124- 3p	Down	p65	Promotion	TNF-α, IL-6, IL-1β	[25]
miR-138	Up	VEGF/NF-κB	Suppression	TNF-α, IL-1β, IL-6 and IL-18	[26]
miR-342-3p	Up	Rictor	Suppression	Foxp3^+^ Regulatory T cells IL-17、IFN-γ and TNF-α	[27]
miR-873a-5p	Up	NF-κB	Suppression	TNF-α, IL-1β, INOS and IL-6	[28]
miR-146a	Up	TLR4/NF-κB	Suppression	TNF-α, IL-6, IL-8 and IL-1 β	[29]
miR-542-3p	Up	TLR4	Suppression	TNF-α, IL-6 and MCP-1	[30]
miR-193b-3p	Up	HDAC3/NF-κB p65	Suppression	IL-1β, IL-6 and TNF-α	[31]
miR-140-5p	Up	HMGB1/PI3K/AKT	Suppression	TNF-α, IL-6, MMP1 and MMP3	[32]
miR-27a-3p	Up	FOXO3/NAPDH/ROS	Suppression	IL-6, IL-8	[33]

“Promotion” indicates that miRNA upregulation or downregulation can promote AP progression. “Suppression” indicates that miRNA upregulation or downregulation can suppress AP progression

## miRNAs and their role in AP progression

**Fig. 1:**
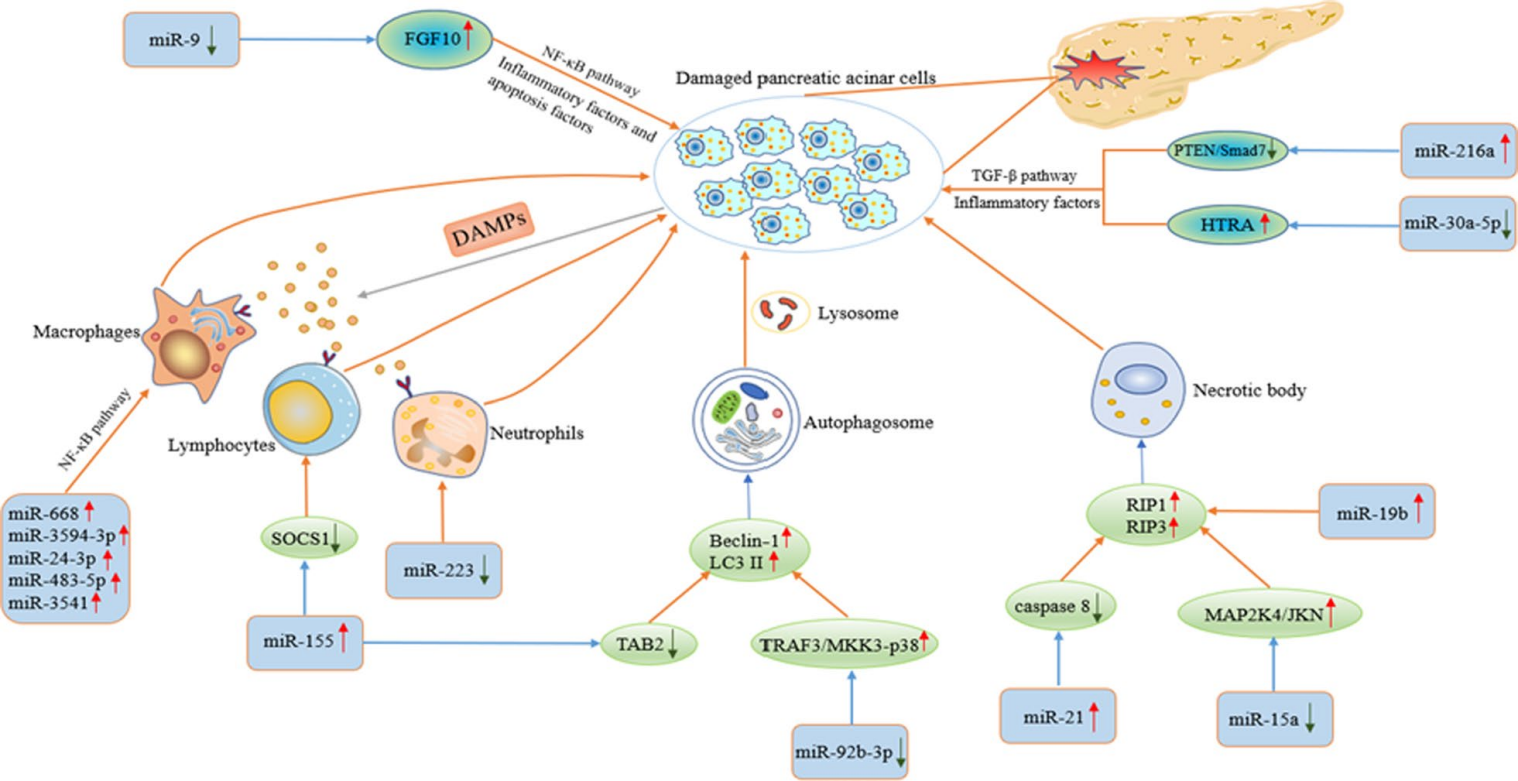
Mechanisms by which miRNAs regulate AP. The figure fully shows the relevant mechanisms by which miRNAs regulate AP, including regulation of inflammatory factors and inflammatory cells through related signal pathways, regulation of AP-related autophagy or necrosis, and promotion of immune cell infiltration and differentiation. These processes ultimately promote damage to pancreatic acinar cells and AP progression

Although most AP patients are mildly ill and the illness is self-limiting, at least 20–30% of AP patients develop SAP within a short period, and better treatments do not exist, ultimately leading to a poor prognosis [8, 34]. Studies have shown that intervention of miRNA-mediated signalling pathways reduces the inflammatory response in AP and the apoptosis of pancreatic acinar cells, which affects the process of AP [35, 36]. This section introduces the research progress in miRNA-related mechanisms in AP. The mechanisms by which miRNAs regulate AP are shown in Fig. 1.

### miRNA/NF-κB and AP

As a stimulating transcription factor, NF-κB regulates immunity, inflammation and other processes that affect cell growth, differentiation and apoptosis, and plays an important role in inflammatory diseases and cancer [37, 38]. Studies have found that NF-κB aggravates AP progression by promoting the transcription of inflammatory cytokines [39]. It is known that miR-9 as a regulator can promote or suppress tumour progression, such as in liver cancer and pancreatic cancer [40, 41]. Previous studies have shown that miR-9 can affect the process of inflammation by regulating NF-κB. In caerulein-treated AR42J cells, the expression of miR-9 decreased, and the levels of the inflammatory factors IL-1β, IL-6 and TNF-α in the cells increased, while Bax and cleaved caspase 3 and 9, which are related to apoptosis, were also upregulated. Overexpression of miR-9 caused a reduction in the levels of the above factors and reduced the inflammation and apoptosis of AR42J cells. Through bioinformatics and dual-luciferase analysis, it was found that miR-9 affected the expression of related inflammatory factors and apoptotic factors by targeting fibroblast growth factor 10 (FGF10) to regulate the NF-κB pathway, thereby weakening the process of caerulein-treated AP [35]. Macrophages related to inflammation can produce various inflammatory mediators and cytokines, such as IL-6, IL-8 and TNF-α, causing damage to local tissues and even organs. The activation of macrophages may play a vital role in the occurrence and development of AP. Previous studies have found that the taurine-induced AP cell model activates the NF-κB and p38 MAP kinase (p38 MAPK) signalling pathways [42]. In this experiment, the supernatant of taurolithocholate (TLC)-treated AR42J cells significantly activated the NF-κB activation level in macrophages. Further studies have shown that miRNAs carried by exosomes or other vesicles are closely related to the activation of macrophages. Through relevant bioinformatics and qRT-PCR analysis, it has been found that differentially expressed miRNAs mainly participate in the activation of macrophages through the TNF receptor-associated factor 6-TGF-beta activated kinase 1/MAP3K7 binding protein 2-transforming growth factor beta activated kinase 1-NF-κB inducing kinase/IκB kinase (TRAF6-TAB2-TAK1-NIK/IKK)-NF-κB pathway, including miR-668, miR-3594-3p, miR-24-3p, miR-483-5p, and miR-3541, but further experiments are needed to verify this hypothesis [43]. In short, targeting miRNA/NF-κB may be able to effectively prevent the process of AP, especially in combination with macrophages and other inflammatory cells, and can provide a new approach for treatment of SAP with systemic inflammatory response syndrome, which merits further research in the future.

### miRNA/TGF-β and AP

Due to the prominent role of TGF-β in inflammation, the immune response, and cell differentiation and proliferation, researchers have begun to extensively study its relationship with AP. Previous studies have reported that inhibiting the expression of TGF-β in an AP mouse model effectively alleviated the progression of AP, providing a theoretical basis for subsequent research [44]. It is known that miR-216a is highly expressed in the pancreas. To explore the specific mechanism of TGF-β in AP, Zhang et al. established a caerulein-induced AP mouse model and found that TGF-β expression was upregulated. After administration of TGF-β inhibitors, the levels of serum amylase, lipase, and the proinflammatory factors TNF-α and IL-6 were significantly lower than levels in the untreated group. Then, different concentrations of TGF-β were used to treat AR42J cells. RT-PCR analysis confirmed that TGF-β increased the expression of miR-216a in a dose-dependent manner, and online prediction tools (Targetscan 5.1, miRanda and PicTar) were used to predict the downstream target genes of miRNA. Further experiments have verified that miR-216a activates the PI3K/AKT and TGF-β signalling pathways through targeted regulation of PTEN and Smad7, and promotes the progression of AP [36]. For a long time, emodin has been widely used in the treatment of AP in China, but its exact mechanism and the target of drug action have not been elucidated. Previous studies have found that high-temperature requirement A (HTRA) can effectively prevent TGF-β1 from becoming mature. The inflammatory signal mediated by HTRA/TGF-β1 may be involved in the process by which pancreatic acinar cells are damaged [45, 46]. Xiang et al. conducted in vivo and in vitro experiments to verify that emodin reduces the number of sodium taurocholate (STC)-treated AP trypsin-positive cells, the release of amylase and the expression levels of the inflammatory mediators TNF-α, IL-6 and IL-1β, and these effects were realized through regulation of HTRA/TGF-β1 signalling pathway. Subsequently, bioinformatics analysis revealed miR-30a-5p as the upstream regulatory molecule of this signalling pathway, and that was verified by experiments such as the dual-luciferase assays detection report [47]. These studies may provide new directions for wide application of emodin and the treatment of AP.

### miRNA/inflammatory cells and AP

Damaged pancreatic acinar cells release inflammatory signals through damage-related molecular patterns (DAMPs) and produce a large number of chemokines, adhesion molecules and cytokines, thereby activating and recruiting a large number of lymphocytes, macrophages and neutrophils. The migration of inflammatory cells to damaged regions further aggravates the inflammatory response [48, 49]. In this process, miRNAs play an important role. Imbalance of the IL-17-producing CD4+ T helper (Th17)/regulatory T (Treg) ratio is related to various autoimmune and inflammatory diseases [50]. An increase in this ratio induces an increase in a large number of cytokines and aggravates the progression of AP. Damage to the pancreas can lead to the accumulation of Th17 cells, forming a vicious cycle. Studies have found that miR-155 can induce inflammatory cells to produce TLR signals and promote systemic inflammation. To further explore the relationship between miR-155 and Th17/Treg ratio, Wang et al. isolated CD4+ T from AP patients, overexpressed miR-155, using flow cytometry found a significant increase in the percentage of IL-17^+^ cells; moreover, downregulation of suppressor of cytokine signalling 1(SOCS1) expression was confirmed via WB, indicating that miR-155 promotes the production of Th17 cells and inhibits the expression of SOCS1. Subsequent use of TargetScan software and dual-luciferase assay verified that SOCS1 is a direct target gene of miR-155 regulates the production of Th17 cells, and in vivo experiments verified the above conclusions. Therefore, targeting miR-155/SOCS1 can effectively interfere with the inflammatory response in AP [51]. In addition, Song et al. found that miR-361-5p can promote Th17 cells to secrete IL-17A and aggravate AP by targeting nuclear factor IA (NFIA) and hes family bHLH transcription factor 1 (Hes1), and these results further deepen the connection between Th17 cells and AP [52]. As previously reported, the activation of macrophages in AP can be mediated by the NF-κB pathway signalling pathway and that the miRNAs involved in this process include miR-668 and miR-3594-3p [43]. At present, there are few studies on the relationship between neutrophils and AP. Dey et al. demonstrated that miR-29a/b1 deletion aggravates pancreatic injury and impairs pancreatic regeneration in AP mice, a result that is consistent with the finding that miR-29a/b1 deficiency causes massive infiltration and activation of inflammatory cells such as neutrophils, and promotes the production of cytokines, such as IL-6, IL-10 and TGFβ1. TGFβ1-mediated pancreatic fibrosis is closely related, but the specific regulatory mechanism still needs to be studied in-depth [53]. The connection between miRNAs and inflammatory cells may effectively interfere with the vicious cycle in AP.

### miRNA/autophagy and AP

As a mechanism to protect cells, autophagy can remove damaged, ageing and nonfunctional organelles or macromolecules, and provide energy for cell growth and proliferation. However, autophagy impairment is closely related to several diseases, such as inflammation, neurodegenerative diseases and tumours [54, 55]. Studies have shown that impaired autophagy is involved in the overactivation of acinar cell trypsinogen, abnormal function of organelles and activation of inflammation in AP, but the specific mechanism remains unclear [56, 57]. Due to the prominent role of miRNAs in AP, it is important to understand whether the pathogenesis of AP involves regulation of the autophagy process. Researchers have observed the effect of miR-155 on AP by injecting AVV-miR-155 and AVV-miR-155 sponges into a caerulein-induced AP mouse model. The results showed that MAP3K7 binding protein 2 (TAB2) expression in pancreatic tissue reduced after the injection of AVV-miR-155, which was in contrast to the results in the AVV-miR-155 sponge group, indicating that miR-155 regulates the expression of TAB2. The increase in TAB2 expression inhibited the increase in Beclin-1 levels and hindered autophagosome formation, while overexpression of miR-155 increased Beclin-1 expression, causing excessive accumulation of p62 and vacuolization in the cytoplasm (increase in microtubule-associated protein light chain 3 (LC3 II) levels), ultimately worsening the degree of autophagy impairment and promoting AP progression. This result was verified in an established SAP mouse model, and knocking down miR-155 significantly reduced the pathological damage to the pancreas and lungs in SAP mice [58]. In recent years, it has been discovered that miR-92b-3p, as a regulatory RNA, participates in a variety of cellular behaviour, including proliferation, migration, apoptosis and autophagy. Sun et al. found that during the formation of autophagosomes, miR-92b-3p inhibits AP autophagy by targeting tumour necrosis factor receptor-associated factor-3 (TRAF3) to regulate the phosphorylated mitogen-activated protein kinase kinase 3 (MKK3)-p38 signalling pathway. In caerulein-treated AR42J cells, the expression level of miR-92b-3p decreased, while the levels of Beclin-1 and LC3 II, which are related to the formation of TRAF3, increased. Knockdown of overexpressed miR-92b-3p or TRAF3 caused a decrease in the expression levels of Beclin-1 and LC3 II. A dual-luciferase assay revealed that miR-92b-3p affects protein translation by binding to the 3'UTR of TRAF3 mRNA and affects the expression of downstream genes. Then relevant molecular biology techniques verified that miR-92b-3p affects the formation of AP autophagosome-related proteins by regulating the TRAF3/MKK3-p38 signalling pathway and inhibits the progression of AP [59]. The regulatory relationship between miRNAs and autophagy provides a theoretical basis for elucidating the pathogenesis of AP and developing therapies targeting autophagy. However further research is still needed.

### miRNA/necrosis and AP

In contrast to apoptosis, necrosis depends on the participation of receptor-interacting protein kinase 1 (RIP1/RIPK1) and receptor-interacting protein 3 (RIP3) and is related to many pathological conditions such as AP, ischaemia–reperfusion injury and neuropathy [60, 61]. Previous studies have found that necrosis is mainly manifested in the high-dose caerulein-induced AP mouse model, but apoptosis is rarely observed. The degree of necrosis is closely related to the severity of pancreatic injury [62]. To study whether oncogenic miR-21 can promote the occurrence of necrosis, researchers established an AP WT mouse model and obtained corresponding pancreatic tissue sections. The results showed that WT mice showed more obvious pancreatic oedema and acinar cell necrosis than the miR-21 konckdown group. The number of CD11b positive cells in the knockdown group was reduced, which significantly affected the infiltration of monocytes and macrophages in the pancreas, and ultimately reduced the severity of AP. Immunofluorescence experiments also confirmed this conclusion. In addition, silencing of miR-21 protects mice from TNF-α-induced systemic inflammatory response syndrome (SIRS), and this process involves silencing of miR-21 to increase the activity of caspase 8, and then downregulating the expression of RIP1/RIP3 to inhibit the formation of microsomes [63]. Hu et al. established an acute necrotizing pancreatitis (ANP) SD rat model. Using miRNA chips and RT-PCR, the expression of miR-19b in ANP was found to be upregulated. This result is consistent with that measured in taurolithocholic acid 3-sulfate disodium salt (TLC-S)-treated AR42J cells. The expression level of miR-19b is positively correlated with the necrosis rate of pancreatic acinar cells, thereby affecting the progression of AP [64]. In another study, baicalin, which has a tumour suppressor effect, significantly reduced the degree of necrosis in AP. This process is achieved through miR-15a targeting of the mitogen-activated protein kinase kinase 4 (MAP2K4)/c-Jun N-terminal kinase (JKN) signalling pathway [65]. The regulatory link between miRNA and necrosis is expected to provide a new therapeutic target for AP. A summary of studies of related miRNAs and their functional roles in the AP process is shown in Table 2.

**Table 2:** Summary of studies of related miRNAs and their functional roles in the AP process

miRNA	Target	Functional role	In vitro/in vivo	References
miR-9	FGF10/NF-κB	Inhibits expression of the inflammatory factors IL-1β, IL-6 and TNF-α, as well as the apoptosis factors Bax and cl-caspase 3/9	In vitro	[35]
miR-668, miR-3594-3p, miR-24-3p, miR-483-5p, miR-3541	TRAF6-TAB2-TAK1-NIK/IKK-NF-*κ*B pathway	Activates macrophages and promotes IL-1β, IL-6 and TNF-α production	In vitro	[43]
miR-216a	Akt and TGF-β Pathway	Promotes TNF-α and IL-6 production	Both	[36]
miR-30a-5p	HTRA/TGF-β1	Inhibits the production of trypsin, amylase and the inflammatory factors TNF-α, IL-6 and IL-1β	Both	[47]
miR-155	SOCS1	Promotes Th17 cell production and IL-6, IL-13 and TNF-α expression	Both	[51]
miR-361-5p	NFIA and Hes1	Promotes Th17 cells to secrete IL-17A	Both	[52]
miR-29a/b1	–	Promotes infiltration of neutrophils and macrophages and release of IL-6, IL-10 and TGFβ1	In vivo	[53]
miR-155	TAB2	Promote the production of Beclin-1 and LC3 II levels and worsens the degree of autophagy damage	In vivo	[58]
miR-92b-3p	TRAF3/MKK3-p38	Increases levels of Beclin-1, LC3 II and autophagosome formation	In vitro	[59]
miR-21	Caspase 8	Upregulation of RIP1 and RIP3 expression promotes necrosome formation	In vivo	[63]
miR-19b	–	Promotes necrosis of pancreatic acinar cells	In vivo	[64]
miR-15a	MAP2K4/JKN	Inhibits the production of IL-1, TNF and IL-6 and reduces pancreatic tissue necrosis	Both	[65]

## miRNA and early diagnosis, severity assessment and prognosis of AP

Early diagnosis and accurate assessment of the severity of a patient's current condition are conducive to the treatment of AP and reduce the incidence of complications and the hospital stay. Although there are currently some biomarkers, such as serum amylase and lipase, and imaging methods (CT, MRI, etc.) for the diagnosis of AP, there is still no single gold standard for predicting the severity of AP [66], especially within 48h after the patient is admitted to the hospital. However, some AP score tables such as the Ranson standard and APACHE II score cannot achieve this goal due to complicated operations. miRNA may become a biomarker for early diagnosis and accurate prediction of the severity of AP due to its key role in the occurrence and development of AP. Liu et al. collected serum samples from 12 AP patients and 3 healthy individuals in Nanchang, China, and performed a microarray analysis of their total miRNAs. They found that there were several differentially expressed miRNAs between SAP and MAP cases, including miR-92b, miR-146b-5p and miR-7. Subsequent RT-PCR analysis quantitatively verified that downregulated miR-92b, miR-10a and miR-7 can be used for the early diagnosis of AP. Moreover, the expression of miR-551b-5p differed significantly between SAP and moderate acute pancreatitis (MAP) patients (p < 0.005), and was correlated with the serum calcium level and complication rate (p < 0.05), indicating that miR-551b-5p is important for predicting the severity of AP [67]. As a serious complication of AP, vascular dysfunction can cause serious organ damage. For this reason, differentially expressed miRNAs reflecting vascular endothelial dysfunction can be used to predict the severity of AP [68, 69]. In another study, by comparing the differential expression profiles of miRNAs in SAP and MAP, it was confirmed that miR-551-5p and miR-126a-5p, which are specifically related to the endothelium, are highly expressed in SAP and are closely related to the severity of AP (AUC 0.716, sensitivity 69.2%, specificity 72.6%, p < 0.001 and AUC 0.748, sensitivity 60.0%, specificity 87.1%, p < 0.001, respectively) [70]. Microarray analysis found that there were significant differences in the expression of serum miRNAs between SAP and moderately severe acute pancreatitis (MSAP) patients with triglycerides. Compared with healthy controls, miR-24-3p, miR-222-3p, miR-361-5p, miR-1246 and miR-181a-5p showed differential expression in the hypertriglyceridemia-induced acute pancreatitis (HTAP) group, and the detection of serum samples revealed that these miRNAs were associated with inflammatory factors (procalcitonin (PCT), IL-1β, IL-6). An ROC working curve confirmed that these miRNAs can accurately assess the progression of HTAP, but further experimental verification is required [71]. Lung injury is a serious complication of SAP, and early prediction is particularly important for improving the prognosis of patients. A bioinformatics analysis performed by Lu et al. found that there were 5 miRNAs (hsa-miR-22-3p, 1260b, 762, 23b and 23a) that were significantly upregulated in SAP patients with acute lung injury (ALI) compared with SAP patients without ALI, and the expression levels of 7 species (hsa-miR-550a*, 324-5p, 484, 331-3p, 22-3p, 140-3p, and 342-3p) were decreased. qRT-PCR verified this result, but the molecular mechanism of regulation still needs in-depth study [72]. In addition, miRNAs also have an important reference value for the prognostic prediction of AP. Li et al. evaluated the value of miR-146a and miR-146b in AP, and found that among patients with SAP, MSAP and MAP, the expression levels of miR-146a and miR-146b were highest in SAP patients and were closely related to the Ranson's score, APACHE II score, SOFA score and C-reactive protein (CRP) level. This increase in miR-146a and miR-146b was accompanied by an increase in the risk of hospital mortality in SAP patients. However, a larger sample size and more data are needed in the future to confirm this finding [73]. In summary, these findings indicate that miRNAs are closely related to the early diagnosis, severity assessment and prognosis of AP and are expected to play an important role in comprehensive treatment of AP. A summary of studies of the miRNA molecular markers related to early diagnosis, prognosis and evaluation of the severity of AP is shown in Table 3.

**Table 3:** Summary of studies of the miRNA molecular markers related to the early diagnosis, prognosis and evaluation of the severity of AP

Molecular marker	Expression	Patient	Clinical relevance	References
miR-92b, miR-10a, miR-7	Up	AP	Diagnosis	[67]
miR-551-5p, miR-126a-5p	Up	AP	Severity assessment	[67, 70]
miR-24-3p, miR-222-3p, miR-361-5p and miR-1246	Up	HTAP	Severity assessment	[71]
miR-181a-5p	Down	HTAP	Severity assessment	[71]
miR-22-3p, 1260b, miR-762, miR-23b and miR-23a	Up	AP with ALI	Diagnosis, prognosis	[72]
miR-550a*, miR-324-5p, miR-484, miR-331-3p, miR-22-3p, miR-140-3p and miR-342-3p	Down	AP with ALI	Diagnosis, prognosis	[72]
miR-146a, miR-146b	Up	AP	Prognosis	[73]
miR-192-5p	Down	AP with NAFLD	Diagnosis	[74]
miR-372	Up	HTGAP	Diagnosis, prognosis	[75]
miR-29a	Up	AP	Severity assessment, prognosis	[76]
miR-7, miR-9, miR-122 and miR-141	Up	AP	Diagnosis, prognosis	[77]
miR-155, miR-21	Down	AP	Diagnosis, severity assessment, prognosis	[78]
miR-127	Down	AP with ALI	Diagnosis, severity assessment, prognosis	[79]
miR-216	Up	AP	Diagnosis, severity assessment	[80]

## miRNA and the treatment of AP

**Fig. 2:**
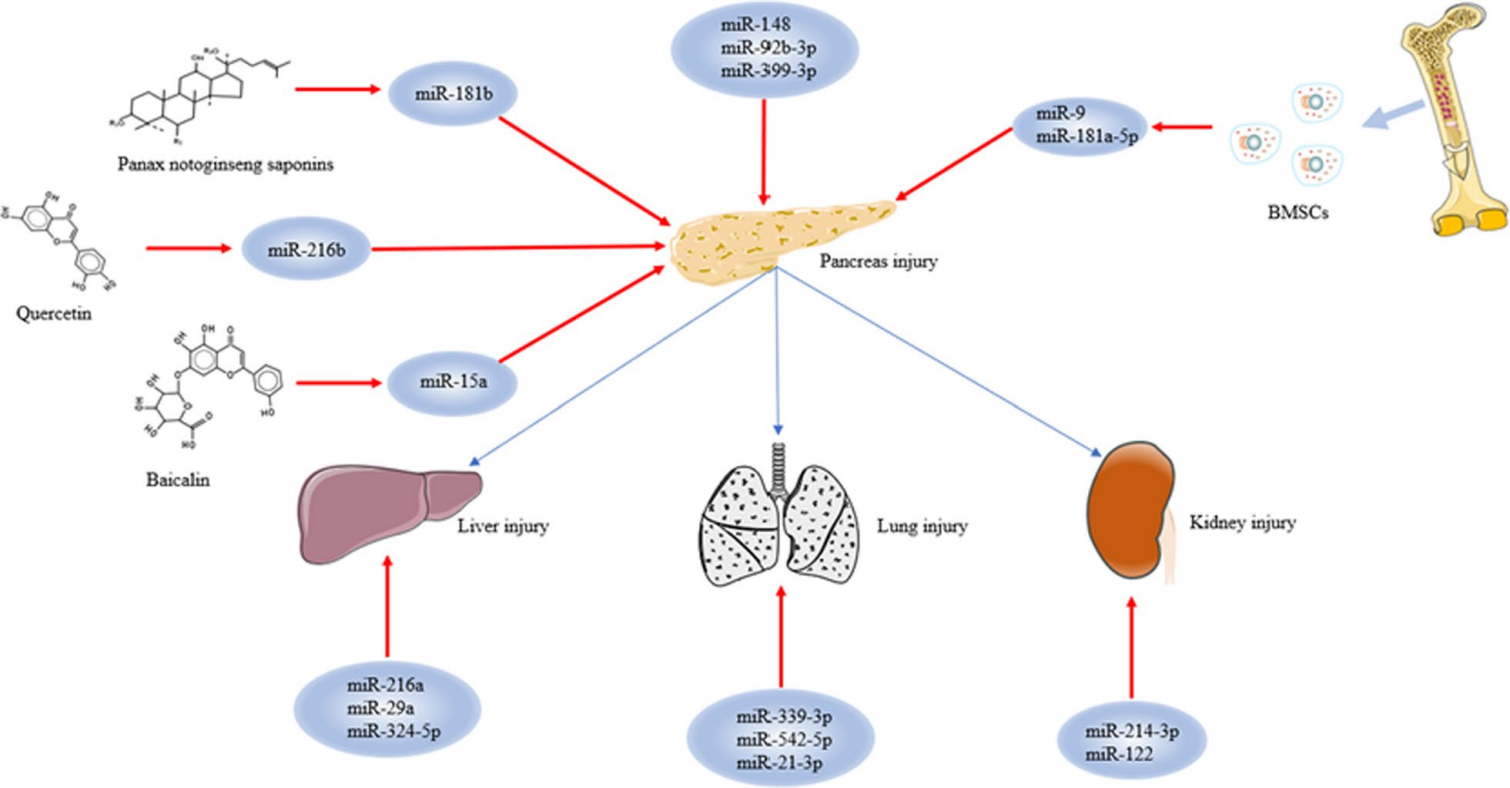
Related miRNAs that could be directly or indirectly targeted for the treatment of AP and its complications. The figure shows that direct targeting of miRNA can attenuate the inflammatory response in AP or, combined with traditional Chinese medicine and MSCs, reverse the severity of AP. For AP with severe complications, therapies targeting miRNAs can also have a good effect

AP is still treated symptomatically. Although measures such as acid suppression, enzyme inhibition, anti-inflammatory drugs and fluid supplementation can play a certain role, their effect is relatively slow. Especially if early intervention is not timely, serious complications and long hospitalization times cannot be avoided [81]. As the roles of individual miRNAs in the progression of AP have been uncovered one by one, especially in the damage associated with distant organs, AP therapy targeting miRNAs has begun to be widely studied. Related miRNAs that could be directly or indirectly targeted for the treatment of AP and its complications are shown in Fig. 2.

### miRNAs and the treatment of simple AP

#### miRNA as a direct target

As a regulatory RNA, the mechanism of miRNAs in the progression of AP provides a research basis for AP-targeted therapies. Abnormal autophagy is an important part of the progression of AP, and how to inhibit this abnormal process has become a research hotspot. Miao et al. established a caerulein-treated AR42J cell model and found that the expression of miR-148a was downregulated. This result also appeared in the pancreatic tissue of AP mice, and the level of cell proliferation increased after overexpression of miR-148a. The corresponding pancreatic tissue was stained with haematoxylin–eosin (HE), and myeloperoxidase (MPO) expression was detected. It was found that the number of MPO-positive cells, the degree of degeneration of acinar cells, the infiltration and necrosis of inflammatory cells, and the vacuole area in cells were decreased in pancreatic tissue overexpressing miR-148a. Subsequent studies found that the expression levels of the autophagy-related proteins LC3-II, Beclin1, autophagy-related gene 4c (ATG4c) and autophagy-related gene 7 (ATG7) decreased with overexpression of miR-148a, and this process was mediated through the IL-6/STAT3 signalling pathway, thereby improving the pathological score of pancreatic tissue in AP mice [10]. miR-92b-3p, which is closely related to cancer, has also been found to play an important role in AP. Researchers established an AP cell model and detected increased expression of the proinflammatory factors TNF-α and IL-6 and the autophagy marker proteins Beclin1 and LC3-II/I. This increase was reversed by overexpression of miR-92b-3p, thereby improving the inflammation and autophagy in AP. Further experiments showed that overexpression of miR-92b-3p caused a decrease in TRAF3, subsequently inhibited the expression of proteins related to the MKK3-p38 signalling pathway (p-MKK3, MKK3, phosphorylated p38 (p-p38) and p38), and finally inhibited the progression of AP [59]. In another study, TRAF3 was shown to be regulated by miR-399-3p. miR-399-3p reduced the expression levels of inflammation-related factors (TNF-α, IL-1β and IL-6) and apoptosis factors (C-caspase3 and Bax), ultimately inhibiting inflammation and apoptosis in caerulein-treated AR42J cells [82]. In addition, Ge et al. found that a decreased abundance of miR-802, which maintains normal pancreatic acinar function, promoted acinar-to-ductal metaplasia (ADM) production and acinar cell proliferation, ultimately causing AP and exacerbating pancreatic injury. However, increasing the expression level of miR-802 effectively inhibited the occurrence and development of this event [83]. These studies provide new potential targets for the treatment of AP.

#### Associated active compounds of traditional Chinese medicine

Based on its unique theoretical system and effective treatment methods, Chinese medicine has become one of the most popular complementary and alternative therapies for the treatment of AP throughout the world. Moreover, increasing number of studies have verified that traditional Chinese medicine (such as curcumin, *Camellia sinensis* and *Zingiber officinale* roscoe) can reduce serum and urine amylase levels, inhibit the production of inflammatory factors, and reduce pancreatic damage. Due to the complexity of traditional Chinese medicine compounds, exploring the active compounds that exert anti-inflammatory effects and their regulatory mechanisms is the main direction of current research on AP therapy, especially combination therapy combined with modern medicine, such as combined miRNA targeted therapy for AP, which has become a research hotspot [84–86]. Panax notoginseng saponins (PNS), which are closely related to oxidative stress, are derived from the extract of Panax notoginseng Ledeb. The antioxidant properties of PNS may be effective in treating AP. Liu et al. established an SAP rat model using taurocholate, and the expression of miR-181b in rats treated with PNS was significantly increased. Subsequently, qRT-PCR and WB showed that the activity of the mammalian target of rapamycin (mTOR)/Akt pathway, which is related to autophagy activation, decreased, while the expression of LC3-II and Beclin1 decreased, leading to a reduction in the number of phagocytes, autophagosomes and autolysosomes. In addition, PNS induced increased apoptosis (increased expression of caspase-3, decreased expression of Blc-2) and significantly improved taurocholate-induced pancreatic injury [87]. Quercetin (QE), which has anticancer and anti-inflammatory effects, has also been shown to improve AP status. Through the establishment of AP cells and mouse models, related experiments have confirmed that QE can reduce the inflammatory factors TNF-α, IL-6 and IL-10. This result is achieved by upregulating miR-216b and inhibiting the MAP2K6/p38 pathway, which ultimately has a protective effect in AP [88]. In addition, the baicalin described above can target the MAP2K4/JKN signalling pathway through miR-15a, which can significantly reduce the necrosis in AP and aid in treatment of AP [65]. The active compounds of these traditional Chinese medicines and miRNAs provide a novel direction for combination therapy in AP.

#### Associated mesenchymal stem cells

As adult stem cells with low immunogenicity, marrow mesenchymal stem cells (MSCs) have the characteristics of self-renewal, immunosuppression, multidirectional differentiation, migration and paracrine activity, and an increasing number of studies have found that they have powerful anti-inflammatory and repair effects [89, 90]. For this reason, the treatment of severe AP with an infusion of MSCs has also attracted much attention. Qian et al. found that the expression of miR-9, which was low in the SAP group, was significantly increased by injection of bone marrow mesenchymal stem cells (BMSCs). The pathological sections also showed that pancreatic oedema, inflammatory infiltration, and necrosis levels decreased after injection of miR-9-modified BMSCs (pri-miR-9-BMSCs) compared with levels in the SAP group. The levels of amylase, lipase and inflammatory factors (TNF-α, IL-1β, IL-6) were all reduced, and pri-miR-9-BMSCs repaired damaged pancreatic tissue by inducing angiogenesis. This process includes promoting the expression of the angiogenesis-related protein angiopoietin-1 (Ang-1), soluble vascular endothelial tyrosine kinase receptor (TIE-2), C-X-C chemokine receptor type 4 (CXCR4) and p-AKT; targeting VE-cadherin and affecting the activity of the β훽-catenin signalling pathway; and recruiting more BMSCs to migrate to damaged tissues, ultimately promoting the regeneration of pancreatic tissue [91]. In another study, it was confirmed that miR-9 can be delivered to the damaged pancreas through pri-miR-9-BMSCs and miR-9 agomir, where it inhibits activation of the NF-κB signalling pathway, reduces proinflammatory factors (TNF-α, IL-1β, IL-6 and HMBG1) and increases the level of anti-inflammatory cytokines (IL-4, IL-10 and TGF-β). This provides an in-depth explanation of the molecular mechanism of AP treatment [92]. In addition, some researchers have found that BMSCs can repair damage in the pancreas by targeting miR-181a-5p. The main pathway involves BMSCs secretion of miR-181a-5p to target PTEN/Akt/TGF-β1 signalling and reduce inflammation and cell apoptosis, ultimately reducing the severity of AP [93]. At present, there is still little understanding of the regulatory mechanism between BMSCs and miRNAs, and further research in the future is expected to provide solid theoretical guidance for combination therapy for AP.

### miRNAs and the treatment of AP with lung injury

If early intervention is not timely, AP will further develop and cause systemic inflammation, leading to serious complications. Lung injury is one of these complications. To explore the role of miRNA in SAP-ALI, Wu et al. established SAP-ALI mouse models and found that the expression of miR-339-3p was reduced in the lung tissue of SAP-ALI mice, while that of Annexin A3 (Anxa3) was the opposite. Bioinformatics predictions and subsequent experiments verified that miR-339-3p reduces inflammation and edema in SAP-ALI mice by targeting Anxa3 to inhibit Akt/mTOR signalling (decreased expression of TNF-α and IL-6) [94]. Some studies have found that miR-542-5p, which is related to late tumour lymphatic metastasis, vascular invasion and TNM staging, is expressed at lower levels in SAP-related ALI mice [95]. To further study the relationship between the two, miR-542-5p was overexpressed in SAP-ALI mice, and the serum amylase, wet-to-dry weight ratio of lung and pancreatic tissue, MPO activity, and severity of pathology all decreased in SAP-ALI mice after miR-542-5p overexpression. This process was mainly caused by a decrease in the expression of related inflammatory mediators and cytokines. The results of dual-luciferase assays revealed that overexpression of miR-542-5p reduced the expression of p21-activated kinase 1 (PAK1), and subsequently inhibited the activation of MAPK-related signalling pathways, including extracellular signal-regulated kinase 1/2 (ERK1/2), JNK and P38MAPK. Finally, miR-542-5p overexpression reduced the release of IL-1β, TNF-α, ICAM-1 and other factors, thereby improving the severity of SAP-ALI [96]. In a study performed by Wang et al., miR-21-3p was found that to be highly expressed in acute haemorrhagic necrotizing pancreatitis (AHNP), and its overexpression activated the transient receptor potential (TRP) signalling pathway; promoted the release of serum amylase, lipase and inflammatory factors; inhibited lung oxygenation; and aggravated pancreatic and lung damage. This result provides a new target for the treatment of SAP-ALI [97].

### miRNAs and the treatment of AP with injury to other organs

Damaged to the liver and kidney is also a common complication of acute pancreatitis, but there are still few studies on the relationship between miRNAs and their interaction. In one study, an HP model complicated with acute renal injury was established through a long-term high-fat diet and sodium taurocholic injection, and serum amylase and blood lipid levels (total cholesterol (T-CHO), triglyceride (TG), low-density lipoprotein cholesterol (LDL-C)) were found to be increased. After miR-214-3p was injected, the expression effect of the above results was enhanced. HE and periodic acid-Schiff (PAS) staining of rat pancreas and kidney tissues revealed pancreatic acinar degeneration, interstitial inflammatory cell infiltration, vasodilation, bleeding, and increased necrosis in the miR-214-3p overexpression group. The degree of swelling of renal tubular epithelial cells and glomeruli was obvious. Sirius red staining revealed a significant increase in the degree of fibrosis in the kidneys and pancreas, while the anti-miR-214-3p group showed the opposite results. These effects were dependent on the activity of the miR-214-3p/PTEN/Akt axis [98]. In addition, studies have found that miR-122 is negatively correlated with the erythropoietin (EPO) level in an induced AP mouse model, and eventually causes anaemia. This may be related to miR-122-mediated kidney inflammatory damage, but relevant experimental verification is needed [99]. At present, there are still few studies on miRNAs as targets for the treatment of SAP complicated by liver injury. In a case–control study, by analysing the peripheral blood of AP patients with or without liver injury, researchers found that elevated miR-216a and miR-29a and reduced miR-324-5p are closely related to the Balthazar CT score, APACHE II score, CRP level and length of stay in the hospital, and the expression level of the above miRNAs can effectively predict the severity of liver injury, but further confirmation is needed [100]. In summary, the above studies clarify the application value of miRNAs in AP with liver and kidney damage and provide a new way to treat severe AP. A summary of studies of the mechanism of action of miRNAs in the treatment of AP and its complications is presented in Table 4.

**Table 4:** Summary of studies of the mechanism of action of miRNAs in the treatment of AP and its complications

miRNA	Mechanism	Disease type	Application	Animal model	References
miR-148a	Inhibits the production of the autophagy-related proteins LC3-II, Beclin1, ATG4c and ATG7 via the IL-6/STAT3 signalling pathway	AP	Suppress	Male BALB/c mice	[10]
miR-92b-3p	Suppresses the expression of proteins associated with the MKK3-p38 signalling pathway (p-MKK3, MKK3, p-p38 and p38) by reducing TRAF3 production	AP	Suppress	–	[59]
miR-399-3p	Inhibits the production of inflammatory and apoptotic factors by regulating TRAF3	AP	Suppress	–	[82]
miR-802	Inhibits ADM production and acinar cell proliferation	AP	Suppress	*mir-802*^* fl/fl*^ mice	[83]
miR-181b	Inhibits autophagy and increases apoptosis by regulating mTOR/Akt when combined with PNS	AP	Suppress	Male Sprague- Dawley rats	[87]
miR-216b	Inhibition of the MAP2K6/p38 pathway when combined with QE	AP	Suppress	C57BL/6 mice	[88]
miR-15a	Combined with baicalin to regulate the MAP2K4/JKN signalling pathway	AP	Suppress	–	[65]
miR-9	Infusion of miR-9-modified BMSCs induces angiogenesis to repair damaged pancreatic tissue	AP	Suppress	Male SD rats	[91, 92]
miR-181a-5p	Regulation of PTEN/Akt/TGF-β1 via secretion of miR-181a-5p from BMSCs	AP	Suppress	Male SD rats	[93]
miR-339-3p	Inhibition of Akt/mTOR signaling by targeting Anxa3	AP with lung injury	Suppress	Male mice	[94]
miR-542-5p	Inhibits the PAK1/MAPK signalling pathway and reduces the release of inflammatory factors	AP with lung injury	Suppress	Mice	[95, 96]
miR-21-3p	Promotes the release of serum amylase, lipase, and inflammatory factors and inhibits pulmonary oxygenation by activating the TRP signalling pathway	AP with lung injury	Promote	Wistar rats	[97]
miR-214-3p	Promotion of pancreatic acinar degeneration and renal tubular epithelial cell swelling via the PTEN/Akt axis	AP with kidney injury	Promote	Male Sprague–Dawley rats	[98]
miR-122	AP promotes the secretion of miR-122 and reduces the level of renal EPO	AP with kidney injury	Promote	C57BL/6 mice	[99]
miR-216a, miR-29a, miR-324-5p	Predicts the severity of liver damage	AP with liver injury	Promote	–	[100]

## Summary and prospects

In recent years, the risk factors for AP have gradually increased, accompanied by high morbidity and mortality. Especially for SAP, it is difficult to avoid serious complications and recurrence with conventional diagnosis and treatment. Therefore, we urgently need to innovate early diagnosis, prognostic evaluation and treatment methods. With extensive research on miRNAs, their unique role in inflammation has laid a solid foundation for in-depth exploration of the regulatory mechanisms involved in the pathogenesis and progression of AP. For example, the upregulation or downregulation of miRNAs affects downstream inflammatory signals to regulate the expression of inflammatory factors and cytokines. In addition, miRNAs can also regulate apoptosis and necrosis-related molecules to promote or inhibit AP process. It is worth noting that the interactions between miRNAs and inflammatory cells, such as recruitment, activation, and induction of differentiation, have improved our understanding of the molecular mechanisms mediated by miRNAs in the pathogenesis of AP, making it possible that miRNAs could be a target for the treatment of AP.

Nevertheless, the current research on the relationship between miRNAs and AP progression is still in the initial stage, such as how miRNAs regulate the activation of trypsinogen, how exogenous miRNAs enter damaged cells through extracellular vesicles, and how miRNAs recruit and activate differentiation-related inflammatory cells. In addition, in future research we should pay more attention to how miRNAs can be used to treat AP, such as how miRNA-related mechanisms highlight the effects of Chinese medicine, how miRNAs can be combined with mesenchymal stem cells to treat SAP with severe complications, such as liver and kidney damage and how miRNAs regulate the activation of immune cells to effectively play immunotherapy role. Although these points will require a long time to be thoroughly studied, miRNA-related therapies can provide new methods and strategies for severe inflammatory diseases such as AP. In short, for targeted treatment of AP, miRNAs have broad application prospects.

## Abbreviations

AP: Acute pancreatitis
SAP: Severe acute pancreatitis
miRNAs: MicroRNAs
PTEN: Phosphatase and tensin homologue
NF-κB: Nuclear factor kappa-B
Wnt/β-catenin: Wnt (wingless) /β-catenin
JAK/STAT: Janus kinase/signal transducer and activator of transcription
TET2/A20: Tet methylcytosine dioxygenase 2/the deubiquitinating enzyme A20
TLR4: Toll-like receptor 4
PDCD4: Programmed cell death protein 4
KLF4: Krüppel-like factor 4
Keap1/Nrf2: Kelch-like ECH-associated protein 1/nuclear factor erythroid 2-related factor 2
IL5RA: Interleukin 5 receptor
VEGF: Vascular endothelial growth factor
HMGB1/PI3K/AKT: High mobility group box 1/phosphatidylinositide 3-kinase/protein kinase B
FOXO3/NAPDH/ROS: Forkhead box O3/nicotinamide adenine dinucleotide phosphate/ reactive oxygen species
FGF10: Fibroblast growth factor 10
p38 MAPK: P38 MAP kinase
TLC: Taurolithocholate
HDAC3: Histone deacetylase 3
TRAF6-TAB2-TAK1-NIK/IKK: TNF receptor-associated factor 6-TGF-beta activated kinase 1/MAP3K7 binding protein 2-transforming growth factor beta activated kinase 1-NF-κB inducing kinase/IκB kinase
HTRA: High-temperature requirement A
STC: Sodium taurocholate
DAMPs: Damage-associated molecular patterns
Th17: IL-17-producing CD4+ T helper
Treg: Regulatory T
SOCS1: Suppressor of cytokine signalling 1
NFIA: Nuclear factor IA
TRAF3: Tumour necrosis factor receptor-associated factor-3
RIP1/RIPK1: Receptor-interacting protein kinase 1
MKK3: Phosphorylated mitogen-activated protein kinase kinase 3
SIRS: Systemic inflammatory response syndrome
ANP: Acute necrotizing pancreatitis
TLC-S: Taurolithocholic acid 3-sulfate disodium salt
MAP2K4: Mitogen-activated protein kinase kinase 4
JKN: C-Jun N-terminal kinase
MAP: Moderate acute pancreatitis
NAFLD: Nonalcoholic fatty liver disease
HTGAP: Hyperlipidaemic acute pancreatitis
HE: Haematoxylin–eosin
MPO: Myeloperoxidase
LC3 II: Microtubule-associated protein light chain 3
ATG4c: Autophagy-related gene 4c
ATG7: Autophagy-related gene 7
PNS: Panax notoginseng saponins
mTOR: Mammalian target of rapamycin
ADM: Acinar-to-ductal metaplasia
QE: Quercetin
MSCs: Mesenchymal stem cells
BMSCs: Bone marrow mesenchymal stem cells
PAK1: P21-activated kinase 1
Anxa3: A higher level of Annexin A3
ERK1/2: Extracellular signal-regulated kinase ½
AHNP: Acute haemorrhagic necrotizing pancreatitis
TRP: Transient receptor potential
PAS: Periodic acid-Schiff
T-CHO: Total cholesterol
TG: Triglyceride
LDL-C: Low-density lipoprotein cholesterol
EPO: Erythropoietin
